# Plasmacytoid, conventional, and monocyte-derived dendritic cells undergo a profound and convergent genetic reprogramming during their maturation

**DOI:** 10.1002/eji.201243106

**Published:** 2013-07-04

**Authors:** Thien-Phong Vu Manh, Yannick Alexandre, Thomas Baranek, Karine Crozat, Marc Dalod

**Affiliations:** 1 Centre d’Immunologie de Marseille-Luminy, UNIV UM2, Aix-Marseille Université, Parc scientifique et technologique de LuminyMarseille, France; 2 Institut National de la Santé et de la Recherche Médicale (Inserm) UMR1104Marseille, France; 3 Centre National de la Recherche Scientifique (CNRS) UMR7280Marseille, France

**Keywords:** Dendritic cell subsets, Gene expression profiling, Human, Maturation, Mouse

## Abstract

DCs express receptors sensing microbial, danger or cytokine signals, which when triggered in combination drive DC maturation and functional polarization. Maturation was proposed to result from a discrete number of modifications in conventional DCs (cDCs), in contrast to a cell-fate conversion in plasmacytoid DCs (pDCs). cDC maturation is generally assessed by measuring cytokine production and membrane expression of MHC class II and co-stimulation molecules. pDC maturation complexity was demonstrated by functional genomics. Here, pDCs and cDCs were shown to undergo profound and convergent changes in their gene expression programs in vivo during viral infection. This observation was generalized to other stimulation conditions and DC subsets, by public microarray data analyses, PCR confirmation of selected gene expression profiles, and gene regulatory sequence bioinformatics analyses. Thus, maturation is a complex process similarly reshaping all DC subsets, including through the induction of a core set of NF-κB- or IFN-stimulated genes irrespective of stimuli.

## Introduction

DCs are specialized in the processing of peptide antigens and their presentation in association with MHC molecules for the activation of naïve Tlymphocytes 1. DCs contribute to preventing autoimmunity by inducing tolerance to self-antigens while promoting immunity against pathogens and cancer. DCs express many innate immune recognition receptors that allow them to sense and integrate microbial, danger, or cytokine signals early during infection or tumorigenesis. During this activation process, DCs undergo morphological, phenotypical, and functional changes that are globally referred to as maturation 2. Mature DCs deliver three kinds of output signals to T cells: (i) cognate engagement of the TCR by MHC+peptide complexes, (ii) engagement of co-receptors by co-stimulation molecules, and (iii) cytokines. Different input signals received by DCs determine the delivery of distinct output signals to T cells which determine their functional polarization 2.

An additional layer of complexity to the functional plasticity of DCs is the existence of different DC subsets that express distinct arrays of innate immune recognition receptors and are specialized in different activities 3. Five major DC subsets exist in mice: plasmacytoid DCs (pDCs), CD8α^+^-type conventional DCs (cDCs), CD11b^+^-type cDCs, monocyte-derived DCs (MoDCs), and Langerhans cells 3. Equivalent DC subsets exist in humans 4–10. pDCs are major producers of the antiviral cytokines type I interferons (IFNs-I) in response to many, although not all, viral infections 11. CD8α^+^-type cDCs encompass lymphoid-tissue resident CD8α^+^ DCs and the CD103^+^CD11b^−^ DCs residing in nonlymphoid tissues but able to migrate to draining lymph nodes upon maturation 12–15. They are especially efficient for CD8^+^ T-cell activation, including through antigen cross-presentation and IL-12 production, hence playing a major role in the induction of cellular adaptive immune responses against intracellular pathogens and tumors 3–15. CD11b^+^-type cDCs encompass lymphoid-tissue resident CD11b^+^ DCs and nonlymphoid tissue CD11b^+^CD207^−^ DCs 15. They are endowed with a high capacity for CD4^+^ T-cell activation and promotion of humoral immunity. pDCs, CD8α^+^-type cDCs, and CD11b^+^-type cDCs constitute a specific lineage of hematopoietic cells derived from a common DC progenitor and depend on the growth factor FLT3-L 15. In contrast, Langerhans cells and MoDCs belong to the monocyte/macrophage lineage and depend on the growth factors GM-CSF or M-CSF 16–17 and IL-34 18, respectively. In vivo inflammation induces the development of a DC subset equivalent to the MoDCs derived in vitro from monocytic precursors in the presence of GM-CSF 10–16. It participates both in direct anti-microbial defense and in adaptive immunity induction 3, especially Th17 induction 10.

Hence, DC maturation must be tightly regulated both by DC subset-intrinsic properties and by the microenvironment in which the cells reside. DC maturation cannot be characterized fully by the measurement of a few parameters, as is generally performed by examining MHC class II molecule export to plasma membrane, co-stimulation molecule upregulation and cytokine production. Large-scale and unbiased approaches should help to better understand the molecular events associated with DC maturation, similar to the advances these approaches brought forward in the characterization of steady-state or inflammatory DC subsets 4–20. Many studies have harnessed the power of gene expression profiling to study the activation of in vitro-derived mouse or human MoDCs, for example to decipher the transcriptional networks mediating their responses to virus-type versus bacterial-type stimuli 21, to investigate kinetically how the induction of their antiviral activity is orchestrated by a network of transcription factors 22 or to examine differences in responses to commensal versus pathogenic fungi 23. Gene expression profiling has also been used to characterize how the artificial loss of the transcription factor TCF4 (E2–2) affects pDC 24. A major reshaping of pDC gene expression program occurred upon loss of E2–2 expression, associated to the acquisition of cDC characteristics and of canonical signs of maturation as occurs upon pDC activation by viral-type stimuli 25–26. Based on these studies, pDCs and cDCs were proposed to fundamentally differ in their physiological responses to activation, pDCs undergoing “a true cell-fate conversion” by differentiating into cDCs as a consequence of a spontaneous decrease in E2–2 expression, while cDCs were thought to be subject to fewer and more subtle changes qualified as “mere maturation” 27. Yet, it is not known whether a true cell-fate conversion of pDCs into cDCs happens under physiological conditions in vivo during a viral infection. Moreover, the extent of the global changes that pDCs and cDCs undergo under the same activation conditions has not been compared. Hence, it is difficult to conclude that activation leads to fundamentally different consequences for pDCs and cDCs. Therefore, we engaged into characterizing how a single physiological perturbation causing the maturation of pDC and cDC in vivo impacts their gene expression programs.

We chose to use mouse cytomegalovirus (MCMV) infection as a working model, because (i) it allows studying the interactions between a virus and its natural host while recapitulating many physiopathological characteristics of the equivalent infection in humans 28, (ii) it leads to the activation of all spleen DC subsets early after intraperitoneal infection, and (iii) all DC subsets contribute to the induction of host immune defenses. We discovered that both pDCs and cDCs undergo profound changes in vivo upon viral infection, encompassing a striking convergence of their gene expression programs through common modulation of hundreds of transcripts.

FLT3-L-dependent DC subsets are now widely accepted to be the most important cell types for the initiation of immune responses in vivo, due to their location in, or efficient migration to, lymph nodes, in contrast to MoDCs which are not present in lymphoid organs under steady-state conditions or only at very low levels and which do not migrate efficiently to lymph nodes 29. It is therefore critical to better understand the molecular mechanisms that regulate the functions of FLT3-L-dependent DC subsets. To contribute to this aim, we provide here data on the gene expression reprogramming of FLT3-L-dependent DC subsets upon their maturation in response to a physiological stimulus. Except for the characterization of the gene expression program of lung CD103^+^CD11b^−^ DCs from mice injected with polyI:C as performed by the Immgen consortium 20, we are not aware of public data on the gene expression profiling of FLT3-L-dependent cDC subsets under microbial-type stimulation conditions, and assuredly not in a context where both cDC subsets and pDCs are studied in parallel. Hence, it was not known to which extent the maturations of pDCs, of FLT3-L-dependent cDCs and of MoDCs were similar, beyond their upregulation of co-stimulation and MHC molecules. We took advantage of our study to address this question by comparing our data with data freely available on public databases, in order to extend our analysis to a variety of stimulation conditions and to other mouse and human DC subsets. We identified, for the first time to the best of our knowledge, a core set of NF-κB- or IFN-stimulated genes induced in DCs upon their maturation irrespective of the stimulus they received, irrespective of the subset they belong to, and conserved in evolution.

## Results

### Mouse cytomegalovirus (MCMV) infection causes a convergent activation of all spleen DC subsets in vivo

**Figure 1:**
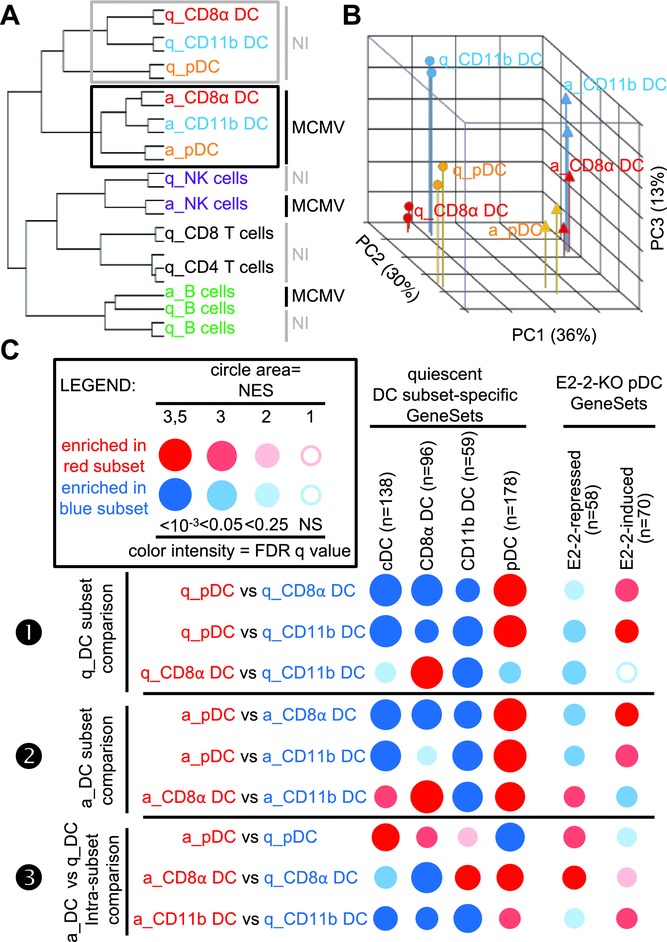
MCMV infection leads to a profound and convergent genetic reprogramming of all splenic DC subsets in vivo. Genome-wide expression analysis was performed on quiescent (q) versus activated (a) immune cells isolated from the spleen of untreated versus d1.5 MCMV-infected C57BL/6 mice. (A) Unsupervised hierarchical clustering of splenocyte subsets from control or MCMV-infected animals was performed. Unsupervised hierarchical clustering with complete linkage was performed on the 28 707 ProbeSets selected as having at least one Log_2_ expression value >5 in the dataset and a fold change >2 across all conditions. Boxes highlight clusters of q_DC subsets (gray) versus a_DC subsets (black). (B) Principal component analysis on all ProbeSets present on the microarrays. (C) DC subsets maintain their identity after activation. GSEA was performed using previously published sets of genes corresponding to the transcriptional fingerprints of q_DC subsets as compared with many other leukocytes 4 or corresponding to the genes up or downregulated in the pDC of E2–2 knock-out mice 32. Pair-wise comparisons were performed to assess enrichment of the GeneSets between 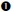 q_DC subsets as a control, 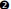 a_DC subsets isolated from d1.5 MCMV-infected mice, and 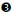 q_DC subsets and their a_DC subset counterparts. Results are represented as circular symbols, the size and intensity of color increasing as the enrichment was stronger and more significant, in a color matching that of the cell subset in which the GeneSet was enriched. Specifically, the circle surface area is proportional to the absolute value of the normalized enrichment score, which varies between ∼1 (no enrichment) and ∼5 (best enrichment possible). The color intensity of circles is indicative of the false-discovery rate statistical q value. Data shown are from two independent replicates from two different experiments except for q_B cells where triplicates were performed and a_B cells where only a singlet experiment was performed.

To assess the general impact of MCMV infection on DC subsets early after virus inoculation, we profiled the gene expression programs of splenic pDCs, CD8α^+^ cDCs, and CD11b^+^ cDCs isolated from control animals (q_cell = quiescent cell) or MCMV-infected mice (a_cell = activated cell). We also included NK cells and B lymphocytes from control versus MCMV-infected animals, and T lymphocytes from control mice. To determine the overall proximity between the cell types isolated from MCMV-infected animals and control mice, we ran an unsupervised hierarchical clustering on the genes that showed at least a twofold change in their expression level across all cell types (Fig. 1A). Two main clusters were obtained: one composed by DCs and the other by lymphoid cells. a_B cells and a_NK cells closely associated with their quiescent counterparts, each of this two lineages constituting a distinct branch within the lymphoid cell cluster irrespective of the activation status of the cells. In contrast, a_DC subsets clustered together, apart from q_DCs. Thus, profound and convergent changes in the gene expression programs of all three splenic DC subsets occurred in vivo early after MCMV infection. Indeed, principal component (PC) analysis showed that “activation versus quiescence” (PC1 axis) and “pDC-ness versus cDC-ness” (PC2 axis) accounted for a similar fraction of the overall variability between the microarrays, respectively 36 and 30% (Fig. 1B). Hence, both the belonging to a given subset and the activation status contributed strongly and equally to the shaping of the gene expression programs of mature DC subsets.

### Activated spleen DC subsets maintain their identity during MCMV infection

We investigated whether each a_DC subset maintained its identity despite their convergent genetic reprograming (Fig. 1C). We previously identified sets of genes specifically expressed in each spleen q_DC subset 4 and addressed here whether these transcriptomic fingerprints were maintained upon activation. We performed Gene Set Enrichment analyses (GSEA) 30 on pairwise comparisons of DC subsets. We generated the control patterns of q_DC subset comparisons (Fig. 1C, 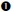) and the patterns of a_DC subset comparisons (Fig. 1C, 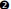). The two figures look remarkably similar, showing that each a_DC subset maintained the transcriptomic fingerprint initially defined in its q_DC subset counterpart. Hence, despite the profound and common changes in gene expression that all DC subsets undergo during MCMV infection in vivo, each activated subset keeps track of its specific lineage through maintained selective expression of a transcriptomic fingerprint, as further illustrated with individual gene expression profiles (Supporting Information Fig. 1A). In particular, pDCs did not undergo a cell-fate conversion into cDCs at 36 hours after MCMV infection at a time when these cells were very close to their maximal upregulation of co-stimulation and MHC class II molecules 31 and when a fraction of them had readily developed dendrites 26.

### Activation downmodulates DC subset expression of their steady-state transcriptomic fingerprint

A comparison of each a_DC subset with its q_DC subset counterpart showed a clear downmodulation of the DC subset-specific transcriptomic fingerprints upon activation (Fig. 1C, 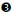 and Supporting Information Fig. 1B). This was not only true for pDCs as predicted 24, but also for CD8α^+^ cDCs and CD11b^+^ cDCs. In addition, the cDC GeneSet was upregulated in a_pDCs as compared with q_pDCs (Fig. 1C, 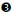, Supporting Information Fig. 1C and 2), and the pDC GeneSet was upregulated in a_CD8α^+^ cDCs and a_CD11b^+^ cDCs as compared with their quiescent counterparts (Fig. 1C, 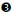, Supporting Information Fig. 1D and 3), contributing to the convergence observed between a_pDCs and a_cDCs in the hierarchical clustering (Fig. 1A). For many genes of the cDC GeneSet, a strong induction was observed in a_pDCs to expression levels similar to those observed in q_cDCs. However, a significant induction also occurred in cDCs leading to expression levels in a_cDCs significantly above those observed in q_cDCs and a_pDCs (Supporting Information Fig. 1C, *Cxcl9*, *Marcksl1*, *Cd86*, *A630077B13Rik*, *Vcam1*, *Traf1*, *Etv3,* and *Aim1*). Of note, *Stx11* and *Trafd1* were strongly induced to the same levels in all DC subsets. Some of the genes that were expressed at higher levels in steady-state pDCs were induced to similar or even higher levels in cDCs upon activation (*Irf7*, *Ly6a*, *Snn,* and *Rilpl1*) or were inversely regulated in a_pDCs and a_cDCs (*Sema4c* and *Serinc5*) (Supporting Information Fig. 1D). However, most of the pDC-specific genes induced in activated cDCs based on GSEA kept much higher levels of expression in pDCs as compared with a_cDCs (as illustrated with *Dirc2*, *Igkc*, *Ly6c1*, *Ctsl*, *Blnk,* and *Klk1* in Supporting Information Fig. 1D). A similar genetic reprogramming occurs in pDCs when they mature after having undergone artificial conditional genetic inactivation of E2–2 32. Therefore, we examined whether the genes known to be regulated by E2–2 in pDCs were affected in DC subsets during their physiological maturation induced by MCMV infection of mice. The “E2–2-repressed GeneSet” regroups the genes induced in pDCs upon genetic inactivation of E2–2 expression 32. The “E2–2-induced” GeneSet regroups the genes inhibited in pDCs upon the conditional knock-out of E2–2 32. Consistent with their natural downregulation of E2–2 (Supporting Information Fig. 1B), a_pDCs showed a significant increase in the expression of the “E2–2-repressed” GeneSet, and, reciprocally, a significant decrease in the expression of the “E2–2-induced” GeneSet (Fig. 1C, 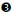 and Supporting Information Fig. 1E and F). Strikingly, the “E2–2-repressed” GeneSet was also significantly upregulated in a_CD8α^+^ cDCs as compared with their quiescent counterpart (Fig. 1C, 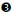, and Supporting Information Fig. 1E), while CD8α^+^ cDCs do not express significant levels of E2–2 (Supporting Information Fig. 1B). Interestingly, while the “E2–2-repressed” GeneSet was globally downregulated in a_CD11b^+^ cDCs as compared with their quiescent counterpart (Fig. 1C, 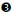), a few of the genes it encompasses were on the contrary induced to high levels in a_CD11b^+^ cDCs as they were in a_pDCs and in a_CD8α^+^ cDCs. This included genes involved in biological processes linked to DC maturation such as dendrite formation for *Fscn1* or cross-talk with CD4^+^ T cells for *Cd40* (Supporting Information Fig. 1E). This contributed to the convergence observed between a_pDCs and a_cDCs in the hierarchical clustering. Hence, not only pDCs but also cDCs downregulate their steady-state transcriptomic fingerprint during viral infection in vivo and upregulate common maturation genes from the “E2–2-repressed” and “cDC” GeneSets, although this may occur in part through different molecular switches in distinct DC subsets.

### Hundreds of genes are modulated in all DC subsets during MCMV infection

**Figure 2:**
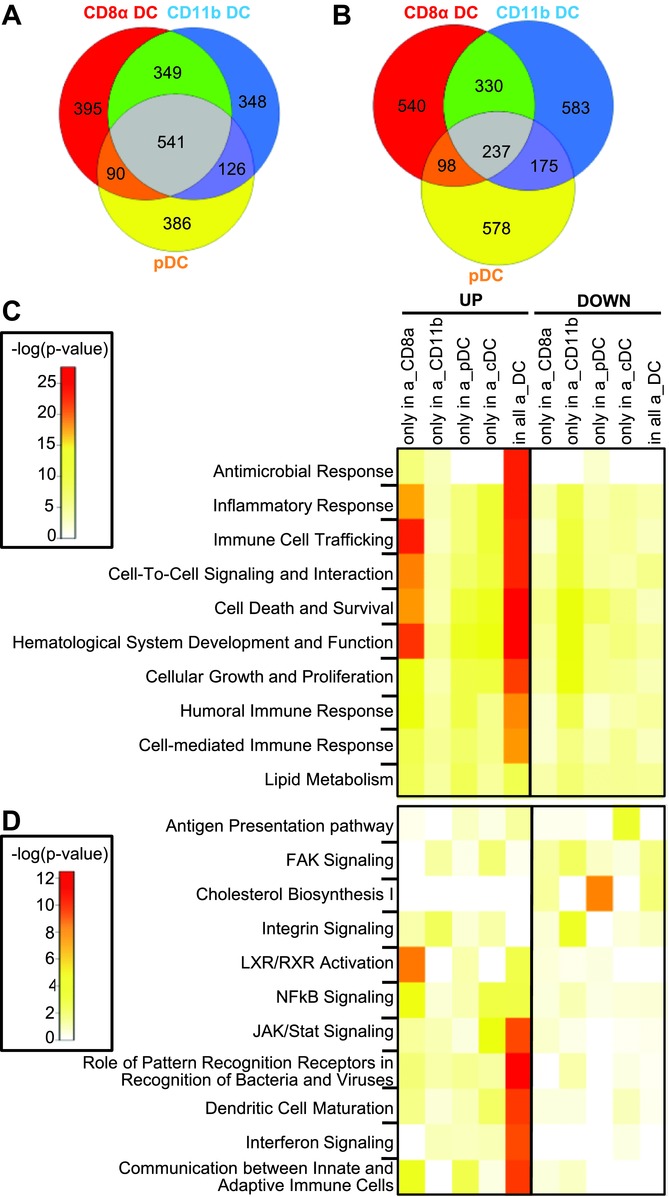
Hundreds of genes are similarly modulated in a_DC subsets as compared with their q_DC subset counterparts. (A and B) The Venn diagrams represent the number of ProbeSets significantly (A) upregulated or (B) downregulated >twofold in a_DC subsets from MCMV-infected mice as compared with their q_DC subset counterparts. (C and D) The heatmaps represent selected functions and pathways found enriched by Ingenuity Pathway Analysis for the sets of genes depicted in the Venn diagrams. The color scale indicates the significance of the enrichment, from white (not enriched) to red (enriched). The data analyzed are the same as in Figure 1.

To compare more precisely how MCMV infection modulates the gene expression program of each of the cell types examined, we identified the ProbeSets that were significantly induced or inhibited ≥twofold in a_DC subsets with respect to their quiescent counterparts. We determined the overlaps between these lists (Fig. 2A and B). 541 Probesets (representing 410 genes) were induced, and 237 ProbeSets (representing 199 genes) were inhibited, commonly in all three DC subsets. Hence, hundreds of genes are commonly modulated in all DC subsets during MCMV infection. Relatively large groups of Probesets were specifically modulated in each DC subset, consistent with the fact that they differed in their capacity to exert specific functions, such as IFN-I production which was much higher in pDCs or IL-15/IL-15Rα expression which was much higher in CD8α^+^ cDCs 11. We examined the annotations of the different groups of genes co-regulated in a_DC subsets, using Ingenuity Pathway Analysis. The most significant functions and pathways found were associated with the genes induced in all a_DC subsets and linked to inflammation, DC maturation, viral sensing, IFN-I induction or signaling, and communication between DCs and NK or T cells (Fig. 2C and D), confirming that, in all DC subsets, maturation significantly enhanced functions critical for cell-intrinsic antiviral defenses and for induction of adaptive immunity.

### A core gene signature is associated with DC maturation upon activation by microbial-type stimuli

**Figure 3:**
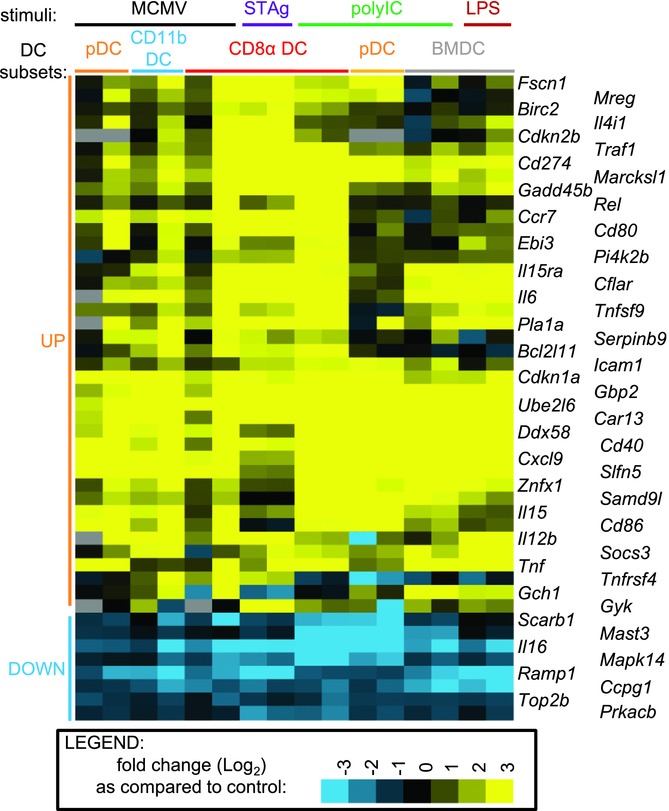
Confirmation of the expression profiles of selected genes by PCR array. The expression pattern of 40 genes upregulated and eight genes downregulated in all splenic DC subsets during MCMV infection as assessed by microarrays was confirmed on independent samples by PCR-array and extended to five other conditions combining different DC subsets (CD8α^+^ cDCs, pDCs, and GM-CSF bone marrow-derived DCs) and different stimuli (in vivo injection of polyIC, in vivo injection of STAg, in vitro stimulation with polyIC or LPS). The data are shown as a heatmap representing the Log_2_ fold change in the expression of each gene in stimulated as compared with unstimulated DCs. Values > = 1 are shown in yellow and values < = −1 in blue. For each activation condition, data shown are from two independent replicates from two different experiments.

**Figure 4:**
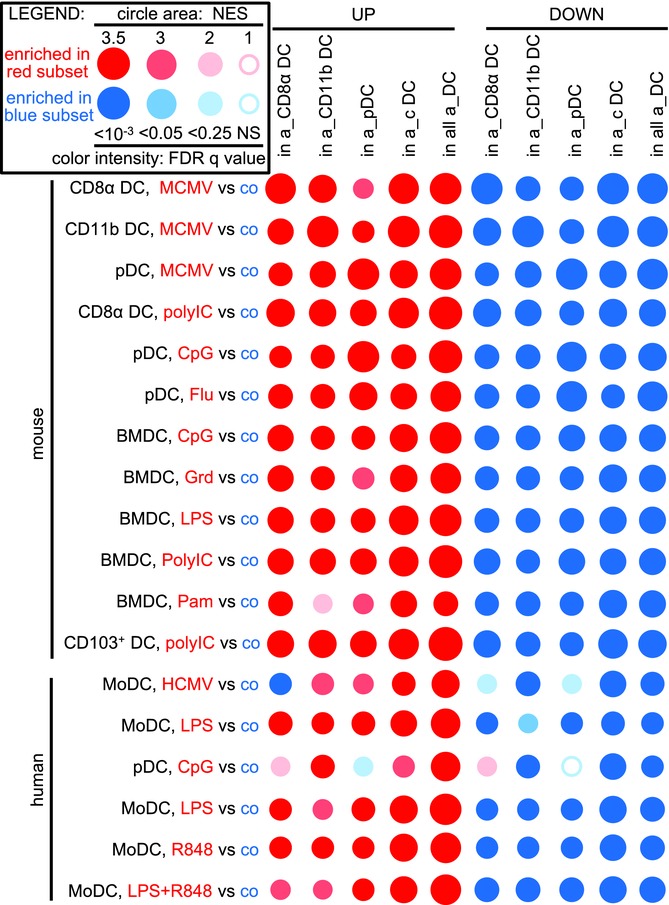
Bioinformatics analysis unraveling a core gene expression program associated with DC maturation conserved between mouse and human irrespective of the microbial stimuli and of the DC subsets studied. GSEA for the Genesets modulated in DC subsets during MCMV infection applied to various public microarray datasets. GeneSets correspond to the different areas of the Venn diagrams in Figure 2. Pair-wise comparisons were performed to assess enrichment of these GeneSets between stimulated versus control cell cultures. The legend is the same as for Figure 1C. The GEO datasets used are listed in Table 1. For each condition, data are shown from pooled replicates of at least two independent experiments.

To test whether the extensive genetic reprogramming of mouse spleen DC subsets observed during MCMV infection could be generalized to other conditions of activation, we examined the expression pattern of a selection of these genes by PCR arrays in different mouse DC subsets upon various stimulations. The transcriptional modulation of these genes during DC maturation was not only confirmed in DC subsets from MCMV-infected mice but also extended to all the other conditions tested (Fig. 3). For further generalization, we analyzed a number of independent public microarray data encompassing gene expression profiling of different mouse or human DC subsets in response to a variety of microbial-type stimuli (Table 1). Specifically, we performed GSEA using as GeneSets the lists of genes modulated in DC subsets during MCMV infection as described in Fig. 2A and B. Strikingly, a significant proportion of the genes modulated in mouse spleen DCs during MCMV infection followed the same pattern of regulation upon DC activation in each of the other conditions examined (Fig. 4). Altogether, when compiling all GSEA performed, 72 genes were significantly upregulated, and 58 genes downregulated, in mature DCs in more than 80% of the 18 different experimental conditions studied, irrespective of stimuli, DC subsets and host species (“core UP” and “core DOWN” genes listed in Table 2 and Supporting Information Table 1). Ingenuity Pathway analysis showed that the genes upregulated in mature DCs were associated with Interferon Signaling (*p* = 1.26 × 10^−8^), NFκB Signaling (*p* = 1.55 × 10^−4^), DC Maturation (*p* = 1.82 × 10^−6^), Communication between Innate and Adaptive Immune Cells (*p* = 4.68 × 10^−6^), Inflammatory Response (*p* = 1.53 × 10^−13^), Antimicrobial Response (*p* = 6.97 × 10^−13^), Hematological System Development and Function (*p* = 1.16 × 10^−20^), Cellular Growth and Proliferation (*p* = 1.16 × 10^−20^), and Cell Death and Survival (*p* = 3.77 × 10^−14^). Fewer annotations were found enriched for the genes downregulated and with much lower statistical significance, such as Notch Signaling (*p* = 8.32 × 10^−3^) and Hematological System Development and Function (*p* = 6.76 × 10^−5^). Finally, using in silico cis-regulatory sequence analyses, we observed a significant enrichment of transcription factor binding sites (TFBS) for NF-κB, ISGF3, IRF8, and IRF7 in the genes upregulated in mature DCs in more than 80% of the conditions examined (Table 3). To functionally test whether a significant proportion of the “core UP” genes with predicted TFBS for ISGF3 or IRF7 required cell-intrisinc IFN-I signaling in DC for their induction, we compared their expression in WT versus *Ifnar1*^−/−^ cDCs from mixed bone marrow chimeric mice. Most of the genes with predicted regulation by IFN-I signaling did require an intact IFN-I receptor in cDCs for their induction during MCMV infection (Supporting Information Table 1). Finally, we investigated to which extent the “core UP” gene expression program from mature DC was specific to the activation of these cells as compared with shared responses with other immune cell types, by analyzing its expression in B cells, NK cells, and CD8^+^ T cells isolated from the same MCMV-infected or control animals. GSEA analysis showed a significant induction of the “core UP” gene set in all the a_cell types examined as compared with their quiescent counterparts, not only in B cells but also in NK cells and CD8^+^ T cells. However, when comparing a_cell types with one another, the “core UP” gene set was significantly induced to higher levels in each activated DC subset as compared with each activated lymphocyte subset (Supporting Information Fig. 4). An analysis of individual genes showed that several genes were consistently induced to a higher level in DCs as compared with the other immune cell types examined, including B lymphocytes (Supporting Information Fig. 5 representative genes in panel 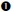 and Supporting Information Fig. 6 cluster 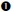). Most of the genes that reached similar levels in activated DCs and in at least another of the cell types examined strongly depended on cell-intrinsic IFN-I signaling for their induction (Supporting Information Fig. 5 representative genes in panel 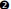 and Supporting Information Fig. 6 cluster 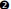) and were generally induced to much lower levels in NK cells as previously reported 33.

**Table 1:** Microarray data used

GEO ID^a^	Cell types^b^	Organism^c^	Stimuli^d^	Time points^e^
GSE21491^f^	CD8α^+^ cDCs, CD11b^+^ cDCs, pDCs, NK cells, B cells	*M. musculus*	MCMV v70 K181	36 h
GSE45365^f^	CD8α^+^ cDCs, CD11b^+^ cDCs, NK cells, CD8^+^ T cells, B cells	*M. musculus*	MCMV v70 K181	36 h
GSE39556^f^	CD8α^+^ cDCs	*M. musculus*	Poly(I:C)	6 h
GSE15907	CD103^+^CD11b^−^ DCs	*M. musculus*	Poly(I:C)	Not available
GSE7831	pDCs	*M. musculus*	CpG 1826; influenza PR8	4 h
GSE17721	BMDCs	*M. musculus*	CpG; LPS; Pam; Poly(I:C); Grd	4 h
GSE10147	pDCs	*H. sapiens*	IL3+CpG	18 h
GSE14000	MoDCs	*H. sapiens*	LPS	4 h
GSE14816	MoDCs	*H. sapiens*	HCMV TB40E	Not available
GSE2706	MoDCs	*H. sapiens*	LPS; R848; LPS+R848	8 h

a Dataset record number in the Gene Expression omnibus (GEO) database.

b Immune cell types studied. MoDCs: human monocyte-derived DCs, derived in vitro in GM-CSF and IL-4; BMDCs: mouse DCs derived in vitro in GM-CSF bone marrow cultures.

c Source organism for the DCs, human (*Homo sapiens*) or mouse (*Mus musculus*).

d Stimuli used to mature the DCs.

e Duration of the stimulation before cell harvest for microarray analysis.

f In-house data.

**Table 2:** List of the genes commonly regulated across DC subsets, stimuli, mouse, and human

Differential expression frequency^a^		Change upon DC maturation	
Human	Mouse	UP	DOWN
6/6	12/12	CCL2, CCL4, CFLAR, CXCL10, GADD45B, GCH1, MARCKSL1, RGS1, RIPK2	CERK, IL16, LYL1, MAPK14, MAST3, MXD4, RAMP1, RCBTB2, TOP2B
6/6	11/12	CD38, GPD2, IFI44, IFIH1, IFIT2, IFIT3, IL15RA, ISG20, MX1, OASL, PML, RSAD2, SERPINB9, TDRD7	CAT, EXOSC5, FES, IFNGR1, INPP5D, LAT2, MICAL1, MYCL1, TK2, CRTAP, CRYL1, FUCA1, TM6SF1
6/6	10/12	CD80, CXCL9, IRF1, OAS2, RTP4, SOS1, STAT1, TAP1, TNFSF10	–
5/6	12/12	CD200, CD86, CLIC4, DNAJA2, IL6, PELI1, REL, SDC4, TANK, TLK2, TMCC3, TNFAIP3, TNFSF9, TRAF1	GLTP, GRK6, HAGH, HMHA1, PDLIM2, PPM1M, PRKDC, RGS18, SFXN3, VPS26B
5/6	11/12	AZI2, B3GNT2, DUSP2, EIF2AK2, GBP2, IL15, IL2RA, NFKBIA, NT5C3, PARP9, PI4K2B, PLA1A, RBBP8, STAT2, TRIM21, UBE2L6, XRN1	ADD3, ARHGAP18, BNIP3L, CYB5R1, HACL1, MFNG, PNPO, SCARB1, ST6GAL1, XPC, ZMAT3
5/6	10/12	CCDC50, CFB, JDP2, LTA, NCK2, NMI, PTGS2, TNFSF4, ZNF281	APEX1, DHRS1, GLUD1, HEXA, HEXB, IDH1, IMPA2, MPHOSPH9, NCOR2, RFNG, RGL2, SMPD2, TRIT1, WDR81, XPOT

a Number of experimental conditions where the genes are found to be significantly modulated in mature DCs as compared to immature DCs/total number of experimental conditions tested.

**Table 3:** TFBS enrichment in the core gene sets modulated upon DC maturation

Expression^a^	Matrix^b^	TF^c^	*p*-Value^d^
UP	IRF_Q6	Irf1; Irf10	<10^−6^
UP	IRF7_01	Irf7a	<10^−6^
UP	NFKB_C; NFKAPPAB65_01		
	NFKAPPAB_01; NFKB_Q6	NFkB	<10^−6^
UP	CREL_01	C-rel	<10^−6^
UP	ISRE_01	Isgf3	7 × 10^−6^
UP	ICSBP_Q6	Irf8	9 × 10^−6^
UP	NFKB_Q6_01	NFkB	1.3 × 10^−5^
UP	IRF1_01; IRF_Q6_01	Irf1; Irf10	<4.7 × 10^−5^
UP	IRF2_01	Irf2	4.7 × 10^−5^
DOWN	USF2_Q6	Usf2a	4 × 10^−6^
DOWN	USF_C	Usf1	9 × 10^−6^
DOWN	NMYC_01	N-myc	2 × 10^−5^
DOWN	MYC_Q2	Myc	4.4 × 10^−5^

a Expression pattern. UP: induced in a_DCs as compared with q_DCs; DOWN: repressed in a_DCs as compared with q_DCs.

b Name of the TFBS matrix as defined in TRANSFAC.

c Transcription factor that could bind to the matrix.

d *p*-Value resulting from the statistical assessment of the enrichment of the matrix in the set of test sequences as compared to the set of control sequences as calculated by using PAASTA (http://trap.molgen.mpg.de/PASTAA.htm).

## Discussion

The goal of the present study was to examine the extent and the specificity of the genetic reprogramming of DC subsets during a viral infection in vivo, to compare the responses between these cell types, and to investigate which signals instruct them. A major aim was to determine whether different DC subsets respond to infection mostly by the modulation of subset-specific gene networks underlying their proposed functional specialization, or whether all DC subsets also share a major transcriptional response.

For the first time to the best of our knowledge, we established that the maturation of spleen-resident DC subsets during a viral infection arose from widespread common changes in their gene expression programs not only in pDCs but also in CD8α^+^ and CD11b^+^ cDCs which were much more profoundly reshaped than anticipated. This partial convergence of the gene expression programs of all the three spleen DC subsets during MCMV infection likely underlies the convergence of their morphological and functional properties as previously reported 25–26. However, despite the extensive reprogramming of their gene expression programs, each DC subset maintained its own cell-type identity even at the mature stage at 36 hours after MCMV infection. Many of the genes specifically expressed in q_pDCs as compared with many other leukocyte types were still expressed to much higher levels in a_pDCs than in a_cDCs, even if they were decreased as compared with those in q_pDCs, including the genes coding for the transcription factors E2–2 and Runx2 and for the specific membrane markers, Siglech and Ccr9. Similarly, a_CD11b^+^ cDCs and a_CD8α^+^ cDCs also kept the expression of their respective specific gene signature defined under steady-state conditions 4. Thus, contrary to what had been proposed 27, the fate of DC subsets during viral infection in vivo cannot be strongly opposed: the changes induced upon maturation are as profound in cDCs as in pDCs, and at least as important as ontogeny in defining the identity of activated DC subsets. In other words, cDCs are as versatile as pDCs since both subsets undergo major and overlapping changes in their gene expression programs upon maturation.

We generalized our study to other DC subsets and conditions of stimulations in both mouse and human. Upon maturation, all mouse and human DC subsets underwent a profound and overlapping genetic reprogramming. We identified 130 genes regulated upon DC maturation irrespective of stimuli and DC subsets and conserved between mouse and human. Their regulatory regions were enriched for putative transcription factor binding sites for ISGF3, IRFs, and NF-κB. Hence, DC maturation must be universally driven by the modulation of a core set of genes controlled by NF-κB, IFN-I, and IFN-γ signaling, in a conserved manner across DC subsets, stimuli, and mammals. In addition to *Cd80* and *Cd86*, several other genes were induced to higher levels in cDC subsets than in B lymphocytes, CD8^+^ T cells and NK cells responding to the same infection in vivo: *Tnfsf9*, *Il6*, *Cxcl9*, *Traf1*, *Marcksl1*, *Pla1a*, *Clic4*, *Il15*, and *Tmcc3*. These genes are therefore likely to critically contribute conferring to mature DCs their primary specific functions, the ability to prime naïve T cells, and to polarize them toward specific functions. While the other genes induced in all DC subsets under all conditions of stimulation were also induced to similar expression levels at least in activated B cells, they are still likely to modulate DC functions. Thus, we believe that our study provides a unique resource for future investigations to continue deciphering the molecular mechanisms modulating DC biology. DC functional plasticity must arise from the superimposition to this core activation pathway of cell subset- and stimuli-specific modules determining in particular the nature of the downstream functional polarization of T cells. The lack of very significant or informative annotations for the genes induced specifically in particular DC subsets upon MCMV infection in vivo emphasizes the need of further studies to better understand the molecular and cellular bases of DC subset-specific functions.

## Materials and methods

### Mice, infection, and in vivo stimulations

8–12 week-old C57BL/6J mice were purchased from Charles River Laboratories and bred at the Centre d’Immunologie de Marseille Luminy (CIML), Marseille, France. Infections were performed by ip injection of salivary gland-extracted MCMV v70 K181 strain (5 × 10^4^ PFU for C57BL/6 animals and 10^4^ PFU for mixed bone marrow chimera mice). 100 μg polyIC or 20 μg STAg were injected iv. Spleen were harvested at 36 hours post-MCMV infection, 3 hours post-polyIC injection, and 12 hours post-STAg injection. Spleen cells were purified as described 4–33. Experiments were conducted in accordance with institutional guidelines for animal care and use (French Provence Ethical Committee Protocols no. 04/2005, 11-09/09/2011, and US Office of Laboratory Animal Welfare Assurance A5665–01).

### Cell sorting and mRNA preparation

Each cell type studied was sorted to over 98% purity (not shown) by flow cytometry using an FACSAria (BD Bioscience) as previously reported 4–33. pDCs were sorted as CD19^−^, CD3^−^, NK1.1^−^, CD11b^−^, 120G8^high^, CD11c^int^ cells. cDCs were sorted as CD19^−^, CD3^−^, NK1.1^−^, 120G8^−/low^, CD11c^high^ and Ly6C^−/low/int^CD11b^+^, or CD8α^+^. Upon MCMV infection, Ly6C is upregulated to some extent on CD11b^+^ DCs but not to the high levels found on monocytes or MoDCs (not shown). Hence, for the purification of spleen CD11b^+^ cDCs, we excluded Ly6C^high^ cells to avoid contamination with monocytes or MoDCs. In consistency, the sorted CD11b^+^ cDCs showed no or only low expression of the *Ly6c1*, *Csf3r*, *Tlr8*, *Cd14*, and *Ccr2* monocyte genes reported to be expressed to higher levels in spleen monocyte-derived CD11b^+^Esam^low^ DCs 34. Duplicate or triplicate samples were generated from independent experiments using pooled spleens of untreated, MCMV-infected, poly(I:C)-injected or STAg-injected mice. High quality total RNA was prepared as described 33.

### Microarray experiments and analyses

Microarray and real-time PCR experiments were performed, processed, and analyzed as described 33. Genes differentially expressed between quiescent and activated DC subsets (Fig. 2A and B) in a statistically significant manner (false discovery rate ≤0.05) were identified using VAMPIRE (http://sasquatch.ucsd.edu/vampire/), a procedure based on a mathematical modeling of the data allowing robust statistical analysis of duplicates 35 and further selected based on a fold change ≥2 as previously published 33. Datasets have been deposited in the GEO database under reference numbers GSE21491, GSE45365, and GSE39556. The core gene signature modulated in >80% of the 18 activation conditions was generated as follows. We applied GSEA to the 18 activation conditions, using as GeneSets the lists of genes modulated in DC subsets during MCMV infection (Fig. 4). On top of the normalized enrichment score and of the significance of the enrichment (false discovery rate), GSEA also provides as an output the leading edge that corresponds to the list of genes that contribute the most to the enrichment score 30. We selected the genes present in at least 10/12 murine and 5/6 human leading edges for each of the GeneSets, and made the union of all these lists for the upregulated genes on one hand, and for the downregulated genes on the other hand (Supporting Information Table 1).

### Real-time PCR experiments and analyses

Real-time PCR experiments and analyses were performed as described 33, using the RT^2^Profiler™PCR Array System with custom-made arrays (SABiosciences).

### In silico cis-regulatory sequence analysis

TFBS enrichment was assessed on the −400 to +400 nt relative to promoter TSS using pattern matching (PASTAA and CLOVER) and motif discovery (MEME) algorithms as previously described 33.

## Abbreviations

a_cell: activated cell
cDC: conventional DC
GSEA: Gene Set Enrichment analyses
IFN-I: type I interferon
MCMV: mouse cytomegalovirus
MoDC: monocyte-derived DC
PC: principal component
pDC: plasmacytoid DC
TFBS: transcription factor binding sites

## Notes

As a service to our authors and readers, this journal provides supporting information supplied by the authors. Such materials are peer reviewed and may be re-organized for online delivery, but are not copy-edited or typeset. Technical support issues arising from supporting information (other than missing files) should be addressed to the authors.
