# Supplementary material for: Plasmacytoid, conventional, and monocyte-derived dendritic cells undergo a profound and convergent genetic reprogramming during their maturation
Source: Eur J Immunol. 2013 Jul 4;43(7):1706–15. doi: 10.1002/eji.201243106 (PMC3799015; doi:10.1002/eji.201243106)
Supplement: Supplementary file 1 [file eji0043-1706-sd1.pdf]

European Journal of Immunology

Supporting Information

for

DOI 10.1002/eji.201243106

Thien-Phong Vu Manh, Yannick Alexandre, Thomas Baranek, Karine Crozat and Marc Dalod

Plasmacytoid, conventional, and monocyte-derived dendritic cells undergo a profound and convergent genetic reprogramming during their maturation

© 2013 The Authors. *European Journal of Immunology* published by Wiley-VCH Verlag GmbH & Co. KGaA Weinheim.

Table S1: List of genes commonly regulated across DC subsets, stimuli, mouse and human

Human Ensembl ID	Human symbol	Mouse symbol	% conditions w/ differential expression[a)]		not enriched in[b)]		Presence of a putative TFBS[c)]				Functional dependency on cell-intrinsic type I IFN signaling: Mean log_2 FC (WT DC) / (Ifnar1$^{-/-}$ DC) from MCMV-infected MBMC mice[d)]	
			human	mouse	human	mouse	NFKB	IRF7	ICSBP	ISRE	in CD8α^{+} cDC	in CD11b^{+} cDC
**Genes up-regulated upon DC maturation**												
ENSG00000163512	*AZI2*	*Azi2*	83%	92%	pDC, CpG vs co	BMDC, Pam vs co					0,35	0,96
ENSG00000170340	*B3GNT2*	*B3gnt2*	83%	92%	MoDC, LPS+R848 vs co	CD103+ DC, PolyIC vs co					0,08	0,23
ENSG00000152492	*CCDC50*	*Ccdc50*	83%	83%	MoDC, HCMV vs co	CD103+ DC, PolyIC vs co; CD8a DC, PolyIC vs co					-0,1	0,22
ENSG00000108691	*CCL2*	*Ccl2*	100%	100%			X				0,16	0,51
ENSG00000129277	*CCL4*	*Ccl4*	100%	100%			X		X	X	0,78	1,45
ENSG00000091972	*CD200*	*Cd200*	83%	100%	MoDC, HCMV vs co						0,45	2,04
ENSG00000004468	*CD38*	*Cd38*	100%	92%		pDC, CpG vs co					-1,29	-0,72
ENSG00000121594	*CD80*	*Cd80*	100%	83%		pDC, CpG vs co; pDC, Flu vs co					0,59	0,22
ENSG00000114013	*CD86*	*Cd86*	83%	100%	pDC, CpG vs co		X				0,15	1,18
ENSG00000243649	*CFB*	*Cfb*	83%	83%	pDC, CpG vs co	BMDC, Pam vs co; BMDC, LPS vs co					0,24	-0,55
ENSG00000003402	*CFLAR*	*Cflar*	100%	100%				X			0,42	0,76
ENSG00000169504	*CLIC4*	*Clic4*	83%	100%	MoDC, HCMV vs co		X				-0,15	0,83
ENSG00000169245	*CXCL10*	*Cxcl10*	100%	100%			X	X		X	1,59	1,25
ENSG00000138755	*CXCL9*	*Cxcl9*	100%	83%		BMDC, Pam vs co; CD8a DC, PolyIC vs co	X	X		X	0,07	0,05
ENSG00000069345	*DNAJA2*	*Dnaja2*	83%	100%	MoDC, LPS vs co					X	-0,32	0,56
ENSG00000158050	*DUSP2*	*Dusp2*	83%	92%	pDC, CpG vs co	CD11b DC, MCMV vs co					0,11	0,08
ENSG00000055332	*EIF2AK2*	*Eif2ak2*	83%	92%	pDC, CpG vs co	BMDC, Pam vs co		X	X	X	2,7	2,41
ENSG00000099860	*GADD45B*	*Gadd45b*	100%	100%			X				1,19	0,74
ENSG00000162645	*GBP2*	*Gbp2*	83%	92%	pDC, CpG vs co	BMDC, Pam vs co					0,07	0,59
ENSG00000131979	*GCH1*	*Gch1*	100%	100%							1,4	1,59
ENSG00000115159	*GPD2*	*Gpd2*	100%	92%		CD8a DC, MCMV vs co					-0,61	0,3
ENSG00000137965	*IFI44*	*Ifi44*	100%	92%		BMDC, Pam vs co		X	X	X	7,69	8,31
ENSG00000115267	*IFIH1*	*Ifih1*	100%	92%		BMDC, Pam vs co	X				3,47	1,73
ENSG00000119922	*IFIT2*	*Ifit2*	100%	92%		BMDC, Pam vs co		X	X	X	6	3,54
ENSG00000119917	*IFIT3*	*Ifit3*	100%	92%		BMDC, Pam vs co	X		X	X	6,27	5,34
ENSG00000164136	*IL15*	*Il15*	83%	92%	pDC, CpG vs co	BMDC, Pam vs co	X	X	X		1,82	3,85
ENSG00000134470	*IL15RA*	*Il15ra*	100%	92%		BMDC, Pam vs co					0,32	0,56
ENSG00000134460	*IL2RA*	*Il2ra*	83%	92%	pDC, CpG vs co	pDC, Flu vs co	X				0,39	0,39
ENSG00000136244	*IL6*	*Il6*	83%	100%	MoDC, HCMV vs co			X	X	X	2,77	0,33
ENSG00000125347	*IRF1*	*Irf1*	100%	83%		BMDC, Pam vs co; pDC, CpG vs co	X				-0,02	0,02
ENSG00000172183	*ISG20*	*Isg20*	100%	92%		BMDC, Pam vs co					5,86	3,81
ENSG00000140044	*JDP2*	*Jdp2*	83%	83%	MoDC, HCMV vs co	BMDC, PolyIC vs co; BMDC, Grd vs co	X		X		0,41	-0,57
ENSG00000226979	*LTA*	*Lta*	83%	83%	MoDC, HCMV vs co	CD103+ DC, PolyIC vs co; BMDC, PolyIC vs co	X				0,58	1,06
ENSG00000175130	*MARCKSL1*	*Marcksl1*	100%	100%							0,94	1,21
ENSG00000157601	*MX1*	*Mx1*	100%	92%		BMDC, Pam vs co		X	X	X	4,86	5,42
ENSG00000071051	*NCK2*	*Nck2*	83%	83%	pDC, CpG vs co	CD103+ DC, PolyIC vs co; CD8a DC, MCMV vs co		X	X	X	0,69	0,34
ENSG00000100906	*NFKBIA*	*Nfkbia*	83%	92%	pDC, CpG vs co	BMDC, PolyIC vs co	X				0,13	0,5
ENSG00000123609	*NMI*	*Nmi*	83%	83%	MoDC, LPS+R848 vs co	BMDC, Pam vs co; BMDC, Grd vs co	X	X			1,05	1,21
ENSG00000122643	*NT5C3*	*Nt5c3*	83%	92%	MoDC, HCMV vs co	BMDC, Pam vs co		X			2,94	3,13
ENSG00000111335	*OAS2*	*Oas2*	100%	83%		BMDC, Pam vs co; BMDC, Grd vs co	X	X	X	X	3,44	3,22
ENSG00000135114	*OASL*	*Oasl1*	100%	92%		BMDC, Pam vs co		X	X	X	4,01	3,92
ENSG00000138496	*PARP9*	*Parp9*	83%	92%	MoDC, HCMV vs co	BMDC, Pam vs co		X	X	X	1,05	1,28
ENSG00000197329	*PELI1*	*Peli1*	83%	100%	pDC, CpG vs co						0,86	0,64
ENSG00000038210	*PI4K2B*	*Pi4k2b*	83%	92%	MoDC, HCMV vs co	BMDC, Pam vs co					0,44	0,5
ENSG00000144837	*PLA1A*	*Pla1a*	83%	92%	pDC, CpG vs co	pDC, Flu vs co	X		X		1,21	0,26
ENSG00000140464	*PML*	*Pml*	100%	92%		BMDC, Pam vs co			X	X	2,32	2,95
ENSG00000073756	*PTGS2*	*Ptgs2*	83%	83%	pDC, CpG vs co	pDC, Flu vs co; CD11b DC, MCMV vs co				X	-1,16	-0,78
ENSG00000101773	*RBBP8*	*Rbbp8*	83%	92%	MoDC, LPS vs co	BMDC, Pam vs co					-0,37	0,03
ENSG00000162924	*REL*	*Rel*	83%	100%	pDC, CpG vs co		X				0,36	0,63
ENSG00000090104	*RGS1*	*Rgs1*	100%	100%			X				1,14	0,48
ENSG00000104312	*RIPK2*	*Ripk2*	100%	100%							0,54	0,26
ENSG00000134321	*RSAD2*	*Rsad2*	100%	92%		BMDC, Pam vs co	X	X			5,2	4,31
ENSG00000136514	*RTP4*	*Rtp4*	100%	83%		BMDC, Pam vs co; BMDC, Grd vs co	X		X	X	4,23	2,89
ENSG00000124145	*SDC4*	*Sdc4*	83%	100%	pDC, CpG vs co		X				-0,65	-1,62
ENSG00000170542	*SERPINB9*	*Serpinb9*	100%	92%		CD103+ DC, PolyIC vs co					-0,11	0,69
ENSG00000115904	*SOS1*	*Sos1*	100%	83%		BMDC, Pam vs co; CD103+ DC, PolyIC vs co			X		0,64	1,17
ENSG00000115415	*STAT1*	*Stat1*	100%	83%		BMDC, Pam vs co; BMDC, Grd vs co					0,7	0,73
ENSG00000170581	*STAT2*	*Stat2*	83%	92%	MoDC, HCMV vs co	BMDC, Pam vs co			X	X	2,65	2,99
ENSG00000136560	*TANK*	*Tank*	83%	100%	pDC, CpG vs co		X				0	0,25
ENSG00000168394	*TAP1*	*Tap1*	100%	83%		BMDC, Pam vs co; CD103+ DC, PolyIC vs co		X		X	0,13	0,4
ENSG00000196116	*TDRD7*	*Tdrd7*	100%	92%		BMDC, Pam vs co	X	X	X	X	1,05	1,91
ENSG00000146872	*TLK2*	*Tlk2*	83%	100%	MoDC, LPS+R848 vs co		X				1,03	1,11
ENSG00000057704	*TMCC3*	*Tmcc3*	83%	100%	MoDC, HCMV vs co						1,59	2,33
ENSG00000118503	*TNFAIP3*	*Tnfaip3*	83%	100%	pDC, CpG vs co		X		X	X	-0,21	0,28
ENSG00000121858	*TNFSF10*	*Tnfsf10*	100%	83%		BMDC, Pam vs co; BMDC, Grd vs co		X	X		1,42	3,4
ENSG00000117586	*TNFSF4*	*Tnfsf4*	83%	83%	pDC, CpG vs co	CD103+ DC, PolyIC vs co; pDC, Flu vs co	X				-0,61	-0,42
ENSG00000125657	*TNFSF9*	*Tnfsf9*	83%	100%	pDC, CpG vs co				X		0,33	0,26
ENSG00000056558	*TRAF1*	*Traf1*	83%	100%	pDC, CpG vs co		X			X	0,92	0,11
ENSG00000132109	*TRIM21*	*Trim21*	83%	92%	MoDC, LPS+R848 vs co	BMDC, Pam vs co		X	X	X	0,47	1,55
ENSG00000156587	*UBE2L6*	*Ube2l6*	83%	92%	MoDC, LPS+R848 vs co	BMDC, Pam vs co	X				1,78	1,64
ENSG00000114127	*XRN1*	*Xrn1*	83%	92%	MoDC, HCMV vs co	BMDC, Pam vs co			X	X	0,89	1,19
ENSG00000162702	*ZNF281*	*Zfp281*	83%	83%	MoDC, LPS vs co	BMDC, Pam vs co; pDC, Flu vs co			X		0,57	0,37
**Genes down-regulated upon DC maturation**												
ENSG00000148700	*ADD3*	*Add3*	83%	92%	pDC, CpG vs co	pDC, CpG vs co			X		-0,71	-1,01
ENSG00000100823	*APEX1*	*Apex1*	83%	83%	pDC, CpG vs co	CD103+ DC, PolyIC vs co; BMDC, Pam vs co					-0,7	-0,94
ENSG00000146376	*ARHGAP18*	*Arhgap18*	83%	92%	MoDC, HCMV vs co	pDC, Flu vs co	X				-0,83	-0,57
ENSG00000104765	*BNIP3L*	*Bnip3l*	83%	92%	MoDC, HCMV vs co	CD103+ DC, PolyIC vs co					-0,28	0,12
ENSG00000121691	*CAT*	*Cat*	100%	92%		BMDC, Pam vs co	X	X			-0,3	0,38
ENSG00000100422	*CERK*	*Cerk*	100%	100%							0,2	-0,6
ENSG00000170275	*CRTAP*	*Crtap*	100%	83%		BMDC, Pam vs co; BMDC, Grd vs co					-0,67	-1,08
ENSG00000165475	*CRYL1*	*Cryl1*	100%	83%		pDC, CpG vs co; CD8a DC, PolyIC vs co	X				-0,44	-0,11
ENSG00000159348	*CYB5R1*	*Cyb5r1*	83%	92%	pDC, CpG vs co	pDC, Flu vs co					-1,15	-1,27
ENSG00000157379	*DHRS1*	*Dhrs1*	83%	83%	pDC, CpG vs co	pDC, Flu vs co; BMDC, LPS vs co					-0,19	-1,05
ENSG00000077348	*EXOSC5*	*Exosc5*	100%	92%		pDC, Flu vs co					-0,07	-0,87
ENSG00000182511	*FES*	*Fes*	100%	92%		CD103+ DC, PolyIC vs co	X	X			0,89	-0,51
ENSG00000179163	*FUCA1*	*Fuca1*	100%	83%		CD103+ DC, PolyIC vs co; pDC, Flu vs co					0,11	-0,35
ENSG00000139433	*GLTP*	*Gltp*	83%	100%	MoDC, LPS+R848 vs co					X	-0,26	0,41
ENSG00000148672	*GLUD1*	*Glud1*	83%	83%	pDC, CpG vs co	BMDC, Pam vs co; pDC, Flu vs co					-0,34	-0,13
ENSG00000198055	*GRK6*	*Grk6*	83%	100%	pDC, CpG vs co					X	0,41	-0,69
ENSG00000131373	*HACL1*	*Hacl1*	83%	92%	MoDC, HCMV vs co	pDC, MCMV vs co					-0,61	-0,45
ENSG00000063854	*HAGH*	*Hagh*	83%	100%	MoDC, HCMV vs co						-0,4	-1,08
ENSG00000213614	*HEXA*	*Hexa*	83%	83%	pDC, CpG vs co	BMDC, Pam vs co; BMDC, Grd vs co			X		-0,15	-1,05
ENSG00000049860	*HEXB*	*Hexb*	83%	83%	MoDC, HCMV vs co	BMDC, Pam vs co; pDC, Flu vs co					-0,27	0,21
ENSG00000180448	*HMHA1*	*Hmha1*	83%	100%	MoDC, HCMV vs co						0,75	0,4
ENSG00000138413	*IDH1*	*Idh1*	83%	83%	pDC, CpG vs co	CD8a DC, MCMV vs co; CD11b DC, MCMV vs co					0,73	0,47
ENSG00000027697	*IFNGR1*	*Ifngr1*	100%	92%		pDC, MCMV vs co	X	X			-0,22	-0,32
ENSG00000172349	*IL16*	*Il16*	100%	100%							0,41	-0,2
ENSG00000141401	*IMPA2*	*Impa2*	83%	83%	pDC, CpG vs co	pDC, MCMV vs co; pDC, Flu vs co					-0,16	-0,95
ENSG00000168918	*INPP5D*	*Inpp5d*	100%	92%		CD103+ DC, PolyIC vs co					-0,08	-0,4
ENSG00000086730	*LAT2*	*Lat2*	100%	92%		pDC, Flu vs co					0	-0,28
ENSG00000104903	*LYL1*	*Lyl1*	100%	100%				X			1,44	-0,23
ENSG00000112062	*MAPK14*	*Mapk14*	100%	100%			X	X	X	X	-0,51	0,63
ENSG00000099308	*MAST3*	*Mast3*	100%	100%							-0,01	-0,68
ENSG00000100060	*MFNG*	*Mfng*	83%	92%	pDC, CpG vs co	BMDC, LPS vs co					0,72	-0,82
ENSG00000135596	*MICAL1*	*Mical1*	100%	92%		pDC, MCMV vs co					0,72	-0,15
ENSG00000051825	*MPHOSPH9*	*Mphosph9*	83%	83%	pDC, CpG vs co	CD103+ DC, PolyIC vs co; pDC, CpG vs co					-0,87	-0,21
ENSG00000123933	*MXD4*	*Mxd4*	100%	100%					X		-0,4	-0,6
ENSG00000116990	*MYCL1*	*Mycl1*	100%	92%		BMDC, PolyIC vs co					0,5	0,33
ENSG00000196498	*NCOR2*	*Ncor2*	83%	83%	MoDC, R848 vs co	CD8a DC, MCMV vs co; CD103+ DC, PolyIC vs co		X	X	X	0,73	-0,9
ENSG00000120913	*PDLIM2*	*Pdlim2*	83%	100%	pDC, CpG vs co						0,15	-0,03
ENSG00000108439	*PNPO*	*Pnpo*	83%	92%	pDC, CpG vs co	pDC, Flu vs co;					0,01	-0,97
ENSG00000164088	*PPM1M*	*Ppm1m*	83%	100%	MoDC, HCMV vs co		X				0,21	-0,8
ENSG00000253729	*PRKDC*	*Prkdc*	83%	100%	pDC, CpG vs co		X				-0,35	-0,04
ENSG00000132329	*RAMP1*	*Ramp1*	100%	100%							-0,68	-0,78
ENSG00000136161	*RCBTB2*	*Rcbtb2*	100%	100%							-0,43	-0,75
ENSG00000169733	*RFNG*	*Rfng*	83%	83%	MoDC, HCMV vs co	CD103+ DC, PolyIC vs co; pDC, Flu vs co					0,55	-0,19
ENSG00000237441	*RGL2*	*Rgl2*	83%	83%	MoDC, LPS+R848 vs co	CD103+ DC, PolyIC vs co; CD8a DC, PolyIC vs co	X	X			0,91	-0,52
ENSG00000150681	*RGS18*	*Rgs18*	83%	100%	MoDC, HCMV vs co						-0,87	-0,71
ENSG00000073060	*SCARB1*	*Scarb1*	83%	92%	pDC, CpG vs co	CD103+ DC, PolyIC vs co;					-0,44	-0,58
ENSG00000107819	*SFXN3*	*Sfxn3*	83%	100%	pDC, CpG vs co						-0,18	-0,42
ENSG00000135587	*SMPD2*	*Smpd2*	83%	83%	pDC, CpG vs co	CD103+ DC, PolyIC vs co; pDC, Flu vs co	X	X	X	X	-0,47	-0,27
ENSG00000073849	*ST6GAL1*	*St6gal1*	83%	92%	pDC, CpG vs co	CD103+ DC, PolyIC vs co;					-0,65	0,13
ENSG00000166548	*TK2*	*Tk2*	100%	92%		pDC, Flu vs co	X				0,66	-0,3
ENSG00000136404	*TM6SF1*	*Tm6sf1*	100%	83%		CD103+ DC, PolyIC vs co; pDC, Flu vs co					-0,64	-0,35
ENSG00000077097	*TOP2B*	*Top2b*	100%	100%			X				-0,49	-0,42
ENSG00000043514	*TRIT1*	*Trit1*	83%	83%	MoDC, HCMV vs co	CD103+ DC, PolyIC vs co; BMDC, LPS vs co					0,33	0,06
ENSG00000151502	*VPS26B*	*Vps26b*	83%	100%	MoDC, HCMV vs co			X			-0,28	-0,7
ENSG00000167716	*WDR81*	*Wdr81*	83%	83%	MoDC, HCMV vs co	BMDC, Pam vs co; BMDC, Grd vs co	X				0,13	-0,11
ENSG00000154767	*XPC*	*Xpc*	83%	92%	MoDC, HCMV vs co	BMDC, Pam vs co					0,49	0,17
ENSG00000184575	*XPOT*	*Xpot*	83%	83%	MoDC, HCMV vs co	CD103+ DC, PolyIC vs co; CD11b DC, MCMV vs co					-0,46	-0,5
ENSG00000172667	*ZMAT3*	*Zmat3*	83%	92%	pDC, CpG vs co	CD103+ DC, PolyIC vs co	X				-0,8	-0,36

[a)] As shown on Figure 4, 6 conditions were tested for human cells and 12 conditions for mouse cells. The % indicated corresponds to the frequency of conditions where the gene was found in the leading edge of the GSEA comparing mature DC to control DC in each species; [b)] list of the conditions in which the gene did not fall into the leading edge of the GSEA; [c)] presence of a putative TFBS as assessed with CLOVER, using a similarity score ≥ 8 between the TFBS matrix and its match in the target gene sequence; [d)] *Ifnar1* $^{-/-}$/WT mixed bone marrow chimeric (MBMC) mice were generated and infected with MCMV for 36 hours or mock treated. WT versus *Ifnar1* $^{-/-}$ CD8α^{+} and CD11b^{+} cDC were isolated from the spleen and used for microarray analyses. The dependency of individual gene regulation on cell-intrinsic type I IFN signaling was assessed by calculating the ratio of expression in WT versus *Ifnar1* $^{-/-}$ DC subsets in each pool of MCMV-infected MBMC mice, for 2 independent pools. The mean log_2 of these two ratio is indicated here. Values near 0 are in white, while values below -1 or above +1 are highlighted in bright blue and bright red, respectively.

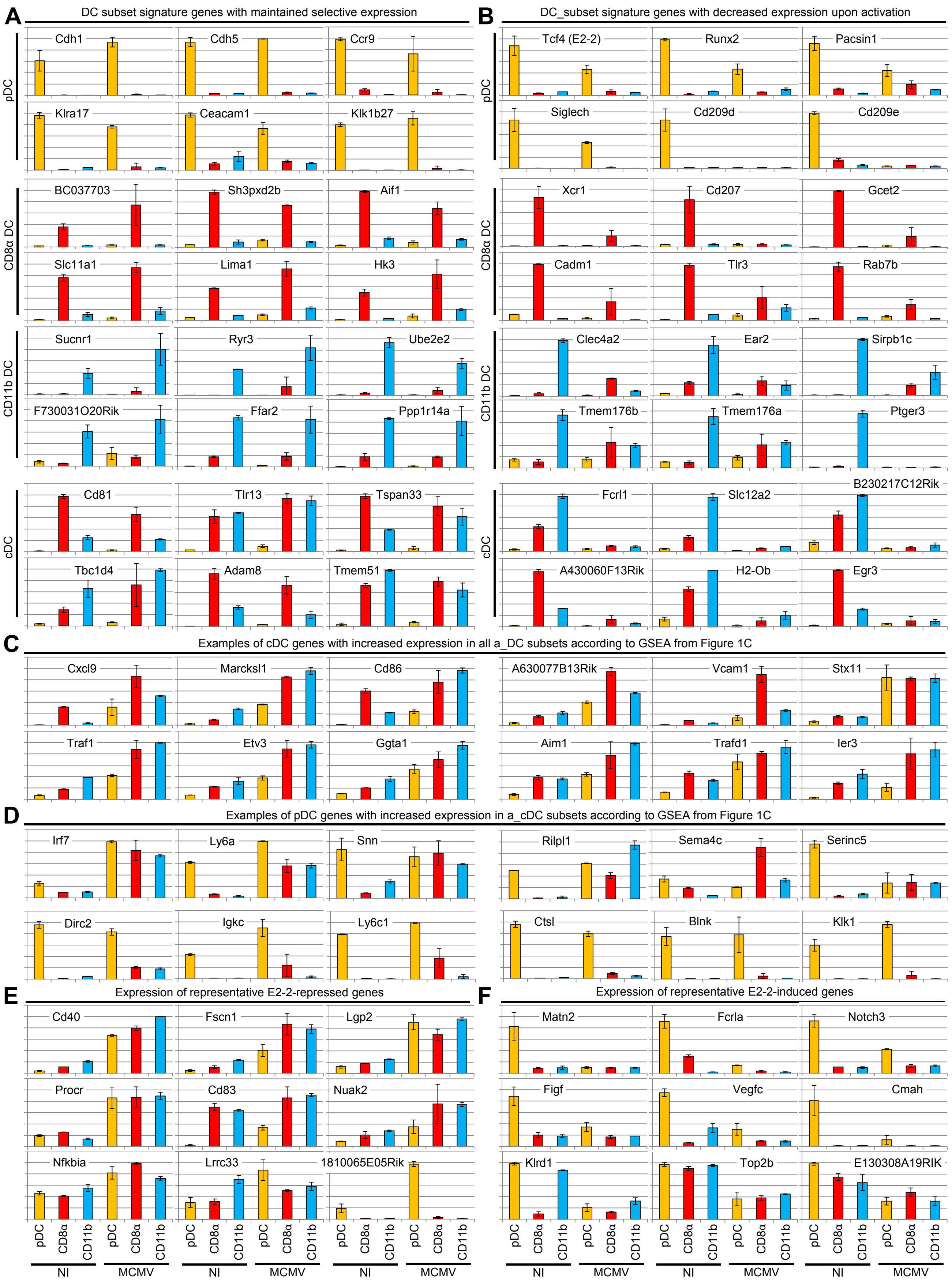

Supporting Information Figure 1. Expression profiles of q_DC subset-specific genes in corresponding a_DC subsets. Data are represented as relative expression levels normalized to maximal expression across the different cell types (Y-axis). Data are shown as mean±SD from 2 independent replicates. (A) Representative genes with maintained selective expression. (B) Representative genes with decreased expression. (C) Genes from the cDC GeneSet with increased expression in all a_DC subsets. (D) Genes from the pDC GeneSet with increased expression in a_cDC subsets. (E-F) Profiles of representative E2-2 target genes in DC subsets.

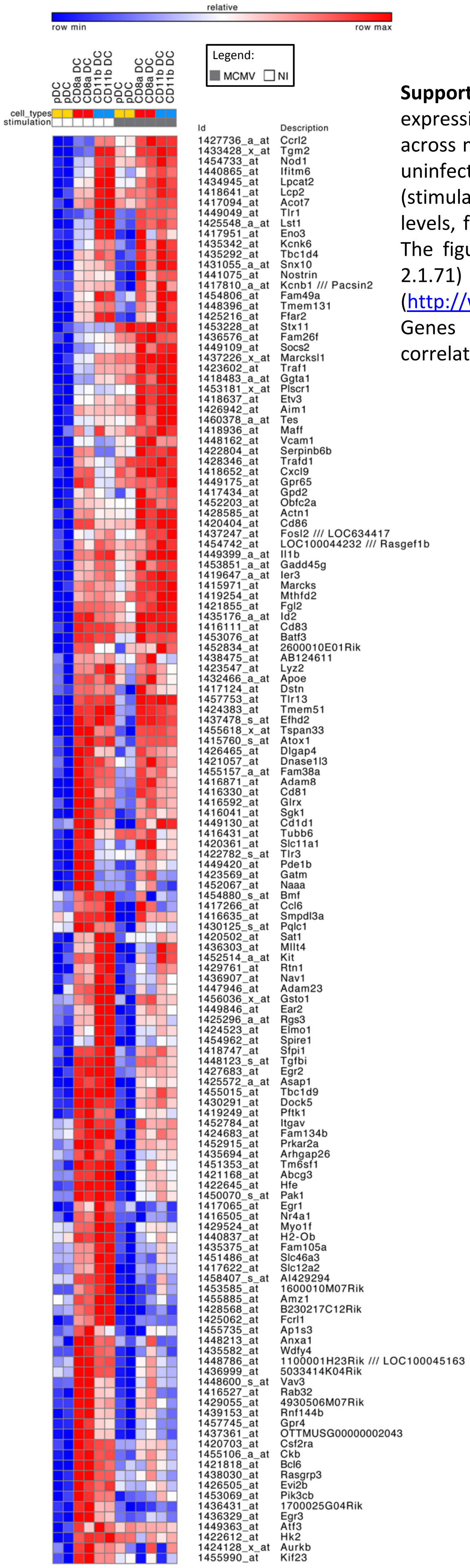

Supporting Information Figure 2: Heatmap of the individual expression profiles of the genes constituting the cDC GeneSet across murine splenic DC subsets (cell types) isolated from mice uninfected (NI) versus infected (MCMV) for 36 hours (stimulation). The color scale indicates the relative expression levels, from blue (lower expression) to red (higher expression). The figure was generated using the software Gene-E (version 2.1.71) from the Broad Institute (http://www.broadinstitute.org/cancer/software/GENE-E/). Genes have been clustered using the "one minus Pearson correlation" distance metric and the Average Linkage method.

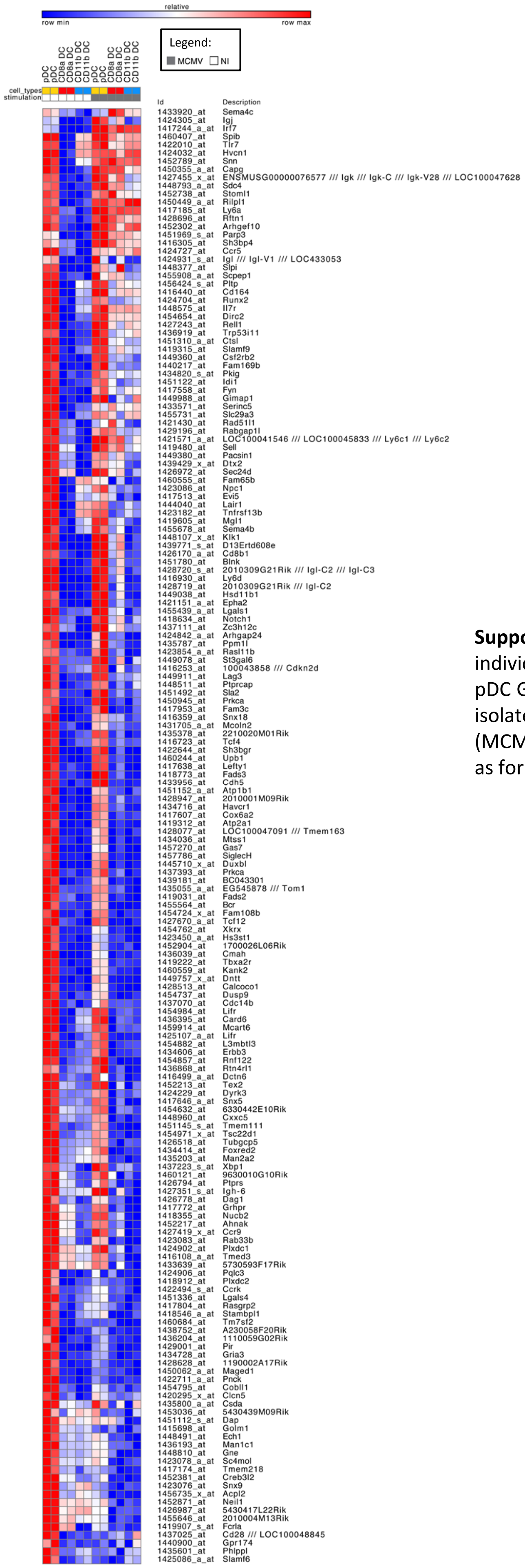

Supporting Information Figure 3: Heatmap of the individual expression profiles of the genes constituting the pDC GeneSet across murine splenic DC subsets (cell types) isolated from mice uninfected (NI) versus infected (MCMV) for 36 hours (stimulation). The legend is the same as for Supporting Information Figure 2.

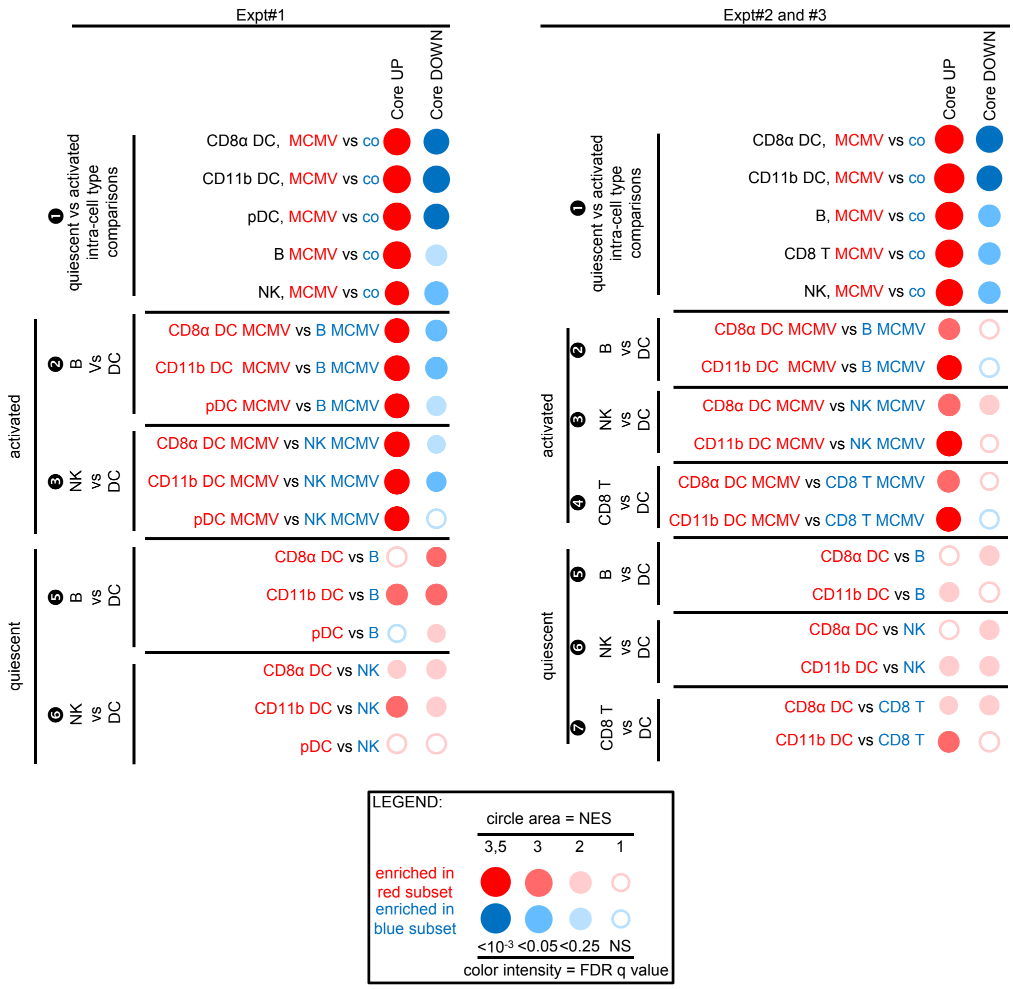

Supporting Information Figure 4: The «core UP» GeneSet induced in mature DC irrespective of subsets, stimuli and species is more strongly up-regulated in DC subsets than in the other immune cells studied in mice infected with MCMV for 36 hours. GSEA using the «core UP» and «core DOWN» GeneSets together with the curated GeneSet collection c2allv3.1 from MSigDB (http://www.broadinstitute.org/gsea/msigdb/index.jsp) was performed on pair-wise comparisons between each activated immune cell type and its quiescent couterpart (❶), activated B lymphocytes versus each activated DC subset (❷), activated NK cells versus each activated DC subset (❸), activated CD8 T cells versus activated cDC subsets (❹), quiescent B lymphocytes versus each quiescent DC subset (❺), quiescent NK cells versus each quiescent DC subset (❻), quiescent CD8 T cells versus quiescent cDC subsets (❼). Three independent experiments were used for these analyses: (A) GSEA performed on experiment #1. (B) GSEA performed on experiments #2 and 3. Experiment#1 was performed in 2004 with C57BL/6 mice. Experiments#2 and #3 were performed in 2011 with mixed bone marrow chimera mice (lethally irradiated CD45.1 C57BL/6 mice reconstituted with a 1:1 mixture of WT cells from CD45.1 C57BL/6 mice and of mutant cells from CD45.2 C57BL/6-*Ifnar1*$^{-/-}$ mice) . The data shown for experiments #2 and 3 are those from the WT cells. The legend is the same as for Fig. 1C.

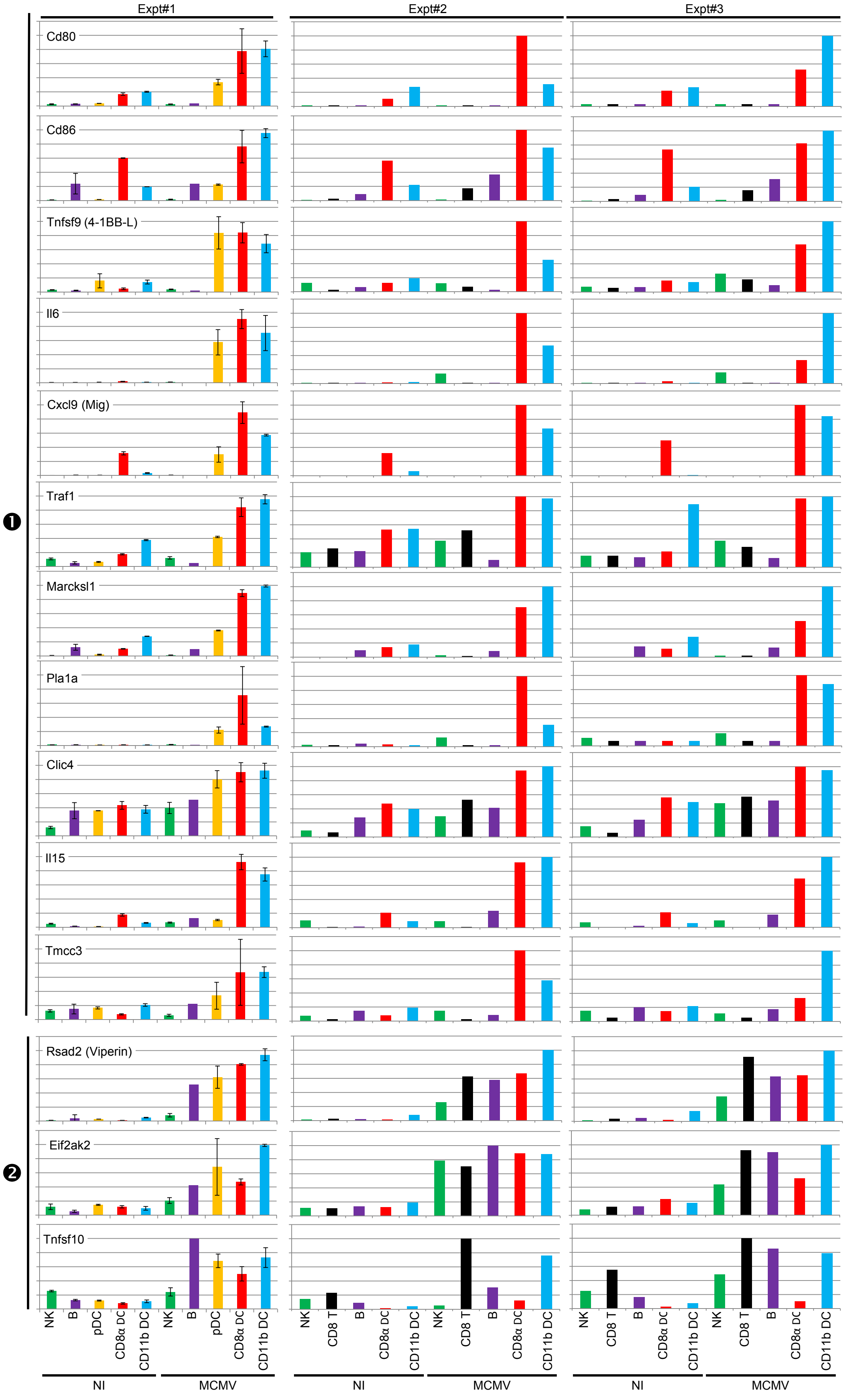

Supporting Information Figure 5. Expression profiles of selected genes from the "core UP" GeneSet across different immune cell types isolated from mice uninfected (NI) or infected (MCMV) in the same 3 independent experiments shown in Supporting Information Figure 4. ❶ Genes induced more strongly in DC than in all other cell types examined. ❷ Examples of genes expressed as strongly in activated B or CD8 T cells as in a_DC and which induction is at least in part dependent on cell-intrinsinc IFNAR signaling. NK= NK cells; B=B lymphocytes; pDC=plasmacytoid dendritic cells; CD8α DC= CD8α^{+} dendritic cells; CD11b DC= CD11b^{+} dendritic cells.

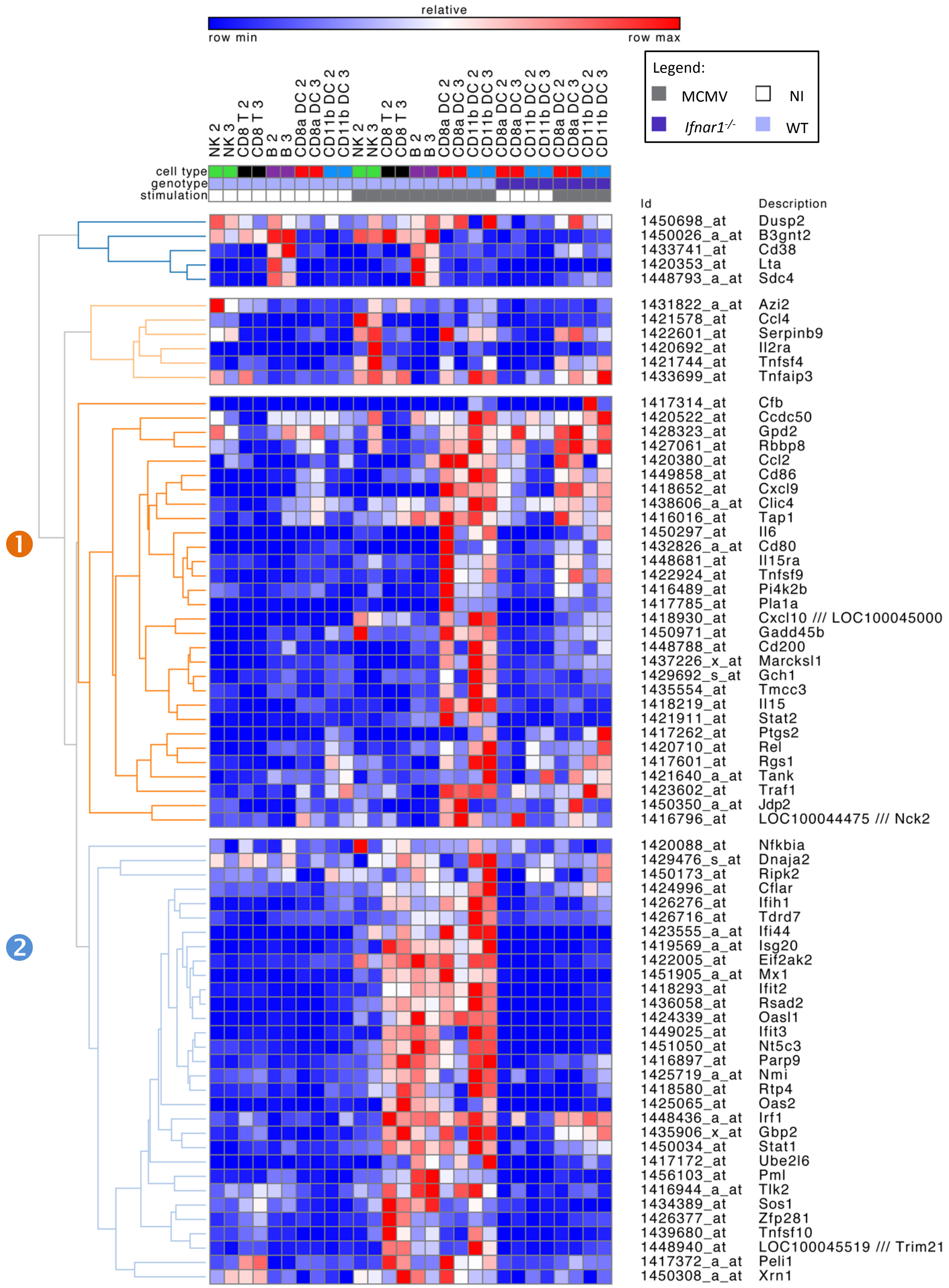

Supporting Information Figure 6: Heatmap of the individual expression profiles of the genes constituting the mature DC «core UP» GeneSet, across all the WT versus *Ifnar1*$^{-/-}$ (genotype) splenocyte populations studied (cell types) isolated from mixed bone marrow chimera mice uninfected (NI) versus infected (MCMV) for 36 hours (stimulation), from two independent experiments (expt#2 and #3 as described in Supporting Information Figure 4). The legend is the same as for Supporting Information Figure 2.
